# Using N400 Event-Related Potential to Detect Differences in Design-Mode and Belief-Mode Scaffold Use

**DOI:** 10.3390/brainsci16040407

**Published:** 2026-04-10

**Authors:** Guangji Yuan, Jumaylha Begum, Rajamanickam Yuvaraj, Chew Lee Teo

**Affiliations:** 1 Centre for Research in Pedagogy and Practice (CRPP), National Institute of Education, 1 Nanyang Walk, Singapore 637616, Singapore; guangji.yuan@nie.edu.sg (G.Y.); chewlee.teo@nie.edu.sg (C.L.T.); 2 Department of Psychology, School of Social Sciences, Nanyang Technological University, 48 Nanyang Avenue, Singapore 639818, Singapore; jumaylha001@e.ntu.edu.sg; 3 Science of Learning in Education Centre (SoLEC), National Institute of Education, 1 Nanyang Walk, Singapore 637616, Singapore

**Keywords:** ERP, scaffolds, design-mode

## Abstract

**Highlights:**

**What are the main findings?**

- A mixed-methods integration of machine learning classification algorithms shows reliable differences between Design-mode and Belief-mode discussion patterns in Knowledge Forum.
- ERP tasks show preliminary evidence consistent with semantic processing differences between scaffolds from Design-mode and Belief-mode, with Belief-mode scaffolded sentences showing larger N400 amplitudes than Design-mode in the 380–430 ms time window, suggesting different semantic processing demands.

**What are the implications of the main findings?**

- Linguistic scaffolds function as “epistemic cues”: Future research can intentionally manipulate sentence starters to experimentally shape epistemic stance in collaborative discussions.
- Scaffold mode modulates semantic processing (N400 window): Belief-mode scaffolded sentences elicited larger N400 negativity than Design-mode, indicating greater semantic conflict. Thus, scaffold choice could shape meaning-making at millisecond timescales even when content is held constant, highlighting the potential value of incorporating Design-mode scaffolds in future learning designs.

**Abstract:**

**Background/Objectives:** Scaffolding plays a vital role in sustaining collaborative discourse and shifting attention. However, current research lacks a detailed understanding of how scaffold use affects participants’ discussions at the neural level. This paper investigates whether epistemic scaffold types (Design-mode and Belief-mode) influence participants’ collaborative discourse and subsequently modulate N400 event-related potential amplitude during sentence processing. **Methods:** Participants in two experimental conditions engaged in an online discussion using scaffolds either representing Design-mode (My theory) or Belief-mode (I agree/I disagree). Participants then individually completed a stimulus-based decision-making task involving sentences representing the two modes. Pre- and post-surveys assessed changes in participants’ attitudes across the study. Machine learning models were used to examine participants’ discourse patterns while event-related potential (ERP) analyses of the N400 component assessed neural responses during the decision-making task. **Results:** Machine learning analyses indicated differences between the two scaffold modes, while ERP analyses revealed a modest N400 amplitude difference between the two modes, during the 380–430 ms time window. **Conclusions:** Findings suggest that epistemic scaffolding can influence collaborative discourse and neural processing, offering implications for the design of scaffolded learning for researchers and practitioners.

## 1. Introduction

Enhancing classroom practices through student engagement is central to learning outcomes. Much learning occurs through social interaction, with language serving as a key mediating tool [1], particularly in discussions that enable the exchange of ideas and the collaborative construction of knowledge. However, little is known about how discussion openings influence the quality and direction of student discourse. Scaffolding strategies have been shown to shift attention and increase brain synchronization, guiding discussions in particular directions [2]. Current understanding, however, remains at the phenomenological level and lacks operationalized linguistic causal input. This study aims to understand how epistemic scaffolds affect group discourse and neural activity across two modes, “Design-mode” and “Belief-mode”.

Design-mode and Belief-Mode: Ideas form the fundamental trajectory of learning, as they are continually developed, refined and improved [3]. In the learning sciences, knowledge-building research identifies two broad approaches to learning: Design-mode and Belief-Mode. Design-mode refers to a wide range of activities credited with knowledge creation and idea improvement, including theorizing, invention, design, identifying promising ideas, and seeking better solutions [4,5]. Belief-mode, on the other hand, refers to the approach in which individuals tend to believe there is only one true answer and evaluate ideas as true and false. This view is derived from the traditional definition of knowledge as justified true belief. Belief-mode includes activities concerning evaluating, questioning, accepting, or rejecting knowledge claims [6]. In the current education system, classroom practices tend to emphasize Belief-mode [5]. In debates or discussions, for example, discourse often stops when certain arguments prevail, perpetuating Belief-mode thinking. Knowledge-building requires a form of discourse that focuses on developing, testing and improving ideas through explanation and theory-building. This is emphasized by Design-mode thinking as introduced earlier. Learners who investigate questions with an open mind in group settings can amplify their understanding by building onto each other’s ideas [7]. Introducing preliminary ideas with sentence starters such as “I don’t understand …”; “I don’t know … but”; “My theory …”; “I found information we should consider …” can play a key role in sustaining ideas and moving the discourse forward [8,9,10].

EEG responses to linguistic cues/scaffolds in education: Linguistic studies have found that recognizing words, retrieving their meanings, and integrating them into discourse are essential to language comprehension. Understanding language and speech requires constructing a series of presentations (phonological, syntactic, and semantic) that link sounds to conversational intention [11]. Research shows that both children and adults engage in top–down processing when listening to information, as their knowledge of the speaker’s intentions and likely sentence meanings shapes their hypotheses about the upcoming words and sounds [11]. When words are presented in the context of a sentence or conversation, higher-level discourse representations constrain lexical access, facilitating the identification of contextually congruent words. Predictable words are therefore recognized more rapidly. From a conversational perspective, listeners’ neural activity couples with that of the speakers’, often in anticipatory ways that predict comprehension and recall [12].

In the EEG literature, the N400 event-related potential (ERP), a negative dip occurring approximately 400 ms after stimulus onset, is widely considered a marker of semantic processing. The N400 is generated in temporal–parietal regions, such as the superior temporal sulcus and the middle temporal gyrus, and is often maximum across centro-parietal scalp sites [13]. Although there are various theories as to its precise role, the N400 is generally associated with semantic processing during language comprehension. For instance, N400 was used as a neural index of semantic congruity during the comprehension of words and sentences [14]. Other studies further used the N400 to understand lexical retrieval [11,15], lexical–semantic processing of new words [16] and predictive processing in monolingual or bilingual comprehension [17,18]. N400 effects have been shown to be sensitive to subtle lexical cues that shape discourse-level interpretation and meaning construction, including pragmatic framing [15,17]. Along similar lines, language that frames ideas as beliefs (e.g., *I think*; *I believe*) may emphasize epistemic stance and personal evaluation, whereas framing ideas as designable objects (e.g., *My idea is*; *This explanation can be improved*) introduces interpretive flexibility and invites elaboration. Taken together, there may be potential value in exploring variations in semantic processing with reference to Design-mode and Belief-mode using N400 responses.

Although previous studies have investigated ERPs in linguistic contexts, less is known about how scaffolds shape the quality of collaborative discourse and information processing at the neural level. This paper aims to explore the following research questions (RQ):

RQ1: How do conversational patterns differ between two experimental conditions using Design-mode and Belief-mode scaffolds?

RQ2: How does the N400 differ between Design-mode and Belief-mode sentence sets during a stimulus-based decision-making task?

RQ3: How do participants’ attitudes differ between Design-mode and Belief-mode conditions in their pre-and post-survey?

## 2. Methodology

Participants: Sixty-six adults (aged 21–44 years; 39 females, 27 males; *M* = 29.20, *SD* = 5.71) participated in the study. Participants were recruited via online community channels and printed study flyers displayed in institutional settings. All participants were right-handed, self-identified as native or fluent English speakers, and had no experience in knowledge-building pedagogy. As the online discussion portion of the study involved collaborative pair work, participants were paired according to scheduling availability. Each pair was then randomly assigned to use either the Design scaffold or the Belief scaffold during the task. The study procedures were approved by the Institutional Review Board (IRB) at Nanyang Technological University (NTU), Singapore (IRB-2024-132, approved [9 July 2025]). Participants provided informed consent before the experiment and were compensated accordingly for their participation. All participants received an honorarium of S$50 for their participation, as approved by NTU-IRB.

Research design and procedure: The study employed a cross-sectional, mixed-methods experimental design. There were five sequential parts to the study: (1) pre-survey, (2) online discussion task, (3) ERP stimulus-based decision-making task, (4) post-survey, and (5) post-experiment interview. Participants were paired only for the online discussion task, during which each pair was randomly assigned to two conditions. All other parts of the study were completed individually during the same session. Upon arrival at the laboratory, participants were seated in separate rooms and were given an overview of the experiment. Thereafter, participants completed the pre-survey. This was followed by approximately 15–20 min of EEG and eye-tracking set-up. Participants then engaged in the online discussion task with their assigned partners. After completing this task, they proceeded to the ERP stimulus-based decision-making task individually. Upon completion, EEG and eye-tracking recordings were stopped. Participants then completed the post-survey. Lastly, participants engaged in a brief individual interview about their overall experience with the online discussion task.

**Figure 1:**
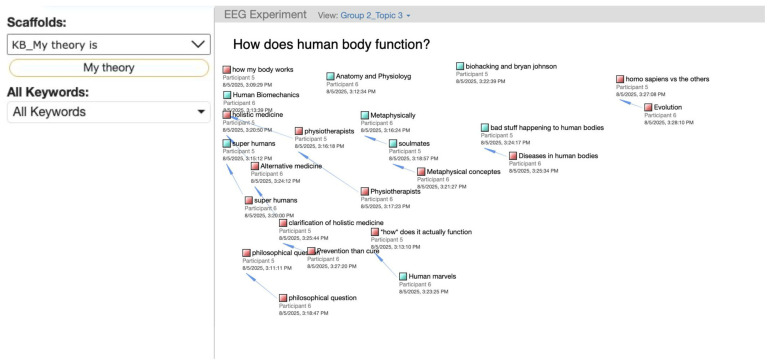
Knowledge Forum discussion with scaffolds. A square dot represents a note posted by the participants, and a blue line indicates the reply and build-on relationship between two notes.

Online discussion task: Participants were seated in two rooms separated by a door, in a laboratory setting at the Science of Learning in Education Centre (SoLEC), National Institute of Education (NIE). The simultaneous discussion task was conducted on Knowledge Forum (KF), an online collaborative learning environment designed to support idea improvement and collective knowledge advancement [19]. KF allows participants to contribute, build-on, and refine ideas within a shared digital space simultaneously (Figure 1). Before beginning the online discussion, participants were individually briefed on how to use the KF platform, including posting new notes, replying to others’ notes, and applying scaffolds. Participants were instructed to post at least two notes per topic, each expressing their own ideas and opinions. They were also asked to reply/build on their partner’s notes, which they would see appear on KF in real time. When replying, participants were required to begin their responses with a designated scaffold, depending on their assigned condition. The pairs in the Design-mode condition were instructed to start their replies with the “My theory” scaffold, while those in the Belief-mode condition started their reply with either “I agree” or “I disagree”, and could use both in the same response if they preferred to do so. Each pair discussed three topics, designed to elicit open-ended reasoning and ideas of elaboration. Topics were chosen based on the science curriculum: (1) How can we have better sustainability? (2) How can we improve education for the 21st century? (3) How does the human body function? Participants were given 20 min per topic, with a 2 min break between topics. Participants were informed that they could refer to online sources (e.g., Google) to support their discussion if needed. EEG and eye-tracking data were recorded continuously throughout this task.

**Figure 2:**
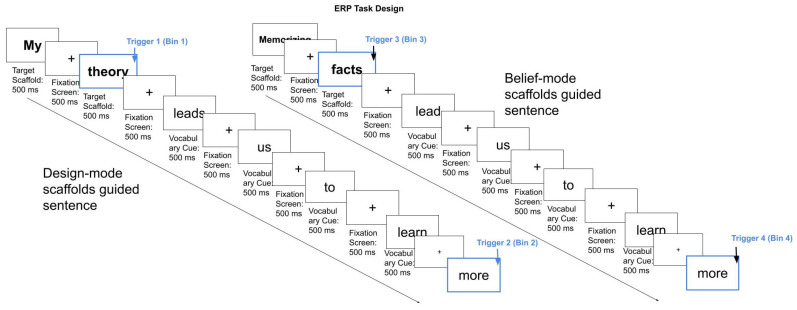
ERP stimuli design: The Design-mode and Belief-mode settings each contain five sentences guided by scaffolds. The four trigger bins were set after the Design-mode scaffolds (Trigger Bin1), at the end of the Design-mode sentence (Trigger Bin 2), at the end of Belief-mode scaffolds (Trigger 3 Bin 3), and at the end of Belief-mode sentence (Trigger Bin4). “+” denotes a fixation cross displayed for 500 ms between each word to centre participants’ gaze before the next stimulus onset.

ERP stimulus-based decision-making task: Two sets of carefully controlled lexical decision-making stimuli were used. Participants were presented with a sequence of short sentences word-by-word on the screen in a randomized order (average of 7–8 words). A total of 10 sentences were presented, with each sentence being repeated 5 times. Given the sensitivity of the ERP measure, scaffold and sentence pairs for each condition were matched for letter length and orthographic neighborhood size. Design-mode scaffolds preceded a phrase (e.g., “*My theory* leads us to learn more”), while Belief-mode scaffolds preceded the same phrase (e.g., “*Memorizing facts* leads us to learn more”). Four triggers were set, including Bin1 which was set after the Design-mode scaffold, and Bin 2 which was set at the end of the Design-mode sentence. For instance, “*My theory* (Bin1) leads us to learn more (Bin2).” In the Belief-mode setting, Bin 3 was set after the Belief-mode scaffolds, and Bin 4 at the end of the Belief-mode sentence. For instance, “*Memorizing facts* (Bin 3) leads us to learn more (Bin 4).” Each word was displayed for 500 ms. Between words, a fixation cross was shown at each point to center participants’ attention for 500 ms (Figure 2). Participants were instructed to indicate whether they agree, disagree, or felt neutral toward each statement using designated keys on a Chronos response device as quickly as possible after each sentence. The task was presented using E-prime version 3.0 software [19] on a 13-inch laptop at the center of the display, approximately 50 cm in front of each participant. EEG and eye-tracking data were recorded continuously throughout this task.

As mentioned previously, language that frames ideas as beliefs may emphasize epistemic stance and personal evaluation, while framing ideas as designable objects may externalize ideas and invite elaboration, thus potentially introducing greater semantic variability and weaker lexical constraints. On this basis, the present study explored if Belief-mode sentence starters would elicit more negative N400 amplitudes than Design-mode sentence starters. Because the sentence starters were embedded in short sentence frames with limited item sets, this task is an initial proof-of-concept. The epistemic scaffold in each pair was designed carefully to represent each mode. However, the verb form immediately following the scaffold is necessarily varied to maintain grammatical coherence (e.g., ‘My theory leads …’ vs. ‘Memorizing facts lead …’). The Design-mode and Belief-mode sentence pairs were carefully matched for overall sentence length, syntactic structure, semantic scenario, and vocabulary. The key manipulation was therefore the scaffold itself, with the remainder of each sentence held as constant as grammatical constraints permitted.

## 3. Data

Knowledge Forum discussion notes: Writing data on KF was used to understand the differences between the two groups’ discourses and collaboration patterns [20]. In total, 66 participants created 1345 notes. With Design-mode, 32 participants formed 16 pairs and wrote 647 notes in total. With Belief-mode, 34 participants formed 17 pairs and wrote 698 notes in total. The Design-mode condition contained 43.80 words per note, whereas the Belief-mode notes contained 41.24 words per note (Table 1).

EEG data acquisition: EEG data were collected using a 64-channel ANT Neuro eego™ system. Signals were sampled at 1000 Hz, and electrode impedances were kept below 20 kΩ throughout the session to the best extent possible and referenced to CPz. EEG data were recorded continuously during the online discussion task and the ERP task stimuli, with event markers synchronized via E-prime. EEG data were processed using MATLAB 2024b and the EEGLAB Toolbox (v2021.1) [21]. Each condition comprises 200 epochs (10 sentences × 5 repetitions × 2 trigger bins). Following the <25% rejection threshold criterion, a minimum of 75 artifact-free epochs per condition were retained per participant.

EEG pre-processing: A total of 14 datasets were excluded due to an insufficient number of artifact-free trials and event-marker synchronization errors for the ERP lexical stimulus-based decision-making task. For the remaining 52 datasets, channels exhibiting excessive noise were identified and interpolated. EEG data were band-pass filtered from 0.1 Hz to 30 Hz, re-referenced to average reference and segmented into bin-based epochs ranging from −200 ms to 1000 ms. Independent Component Analysis (ICA) was conducted to remove eye movement, eye blinks, and muscle-related artifacts. ERPs were obtained by time-locking to trigger markers (Bin 1–4) and averaging across trials within each condition. The sentence content remained identical across repetitions, with only the presentation order randomized.

**Figure 3:**
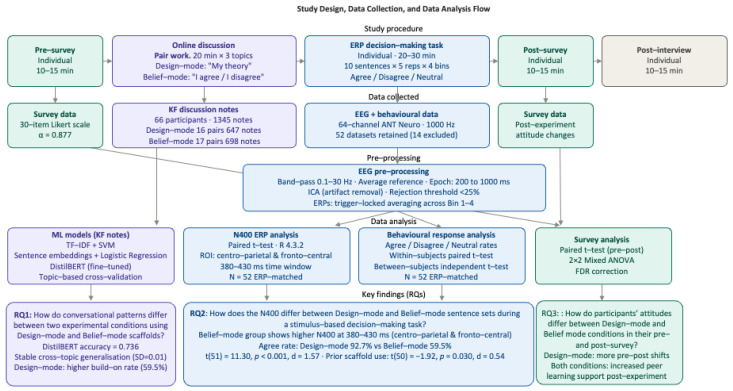
Research design, data collection, and data analysis flow.

Pre- and post-survey: The survey aimed to examine participants’ knowledge-building attitudes with 30 items in total and key constructs including collaborative responsibility, collaboration, and creativity. All items were rated on a 5-point Likert scale. The survey reflected reliable internal consistency, Cronbach’s α = 0.877, indicating a consistent set of operationalized items (Figure 3).

## 4. Data Analysis

Machine learning and Knowledge Forum notes: To determine whether there were differences between the two groups that used Design- and Belief-mode scaffolds, we applied machine learning models to detect them. Learning discourses in KF contain Design and Belief epistemic stances, so we applied computational models to infer latent frames in participants’ online discourses using a multi-mode approach, including (1) lexical weighting models to understand the lexical patterns, (2) embeddings that encode semantic similarity, and (3) transformers to investigate contextualized meanings and context-dependent epistemic framing (Table 2). To investigate lexical patterns, we first applied TF-IDF [22] and a linear SVM [23]. TF-IDF converts each note into weighted word vectors, and the linear SVM trains a linear classifier to distinguish between two-class pattern recognition problems, providing feature weights and lexical contribution to the classification. The weights show the importance of each term across the corpus. Second, to further examine the semantic levels of epistemic frames, which reflect meaning similarity across notes, we applied semantic embeddings [24] and logistic regression as a binary classification. Sentence embeddings map each note into a dense semantic vector as a fixed-length representation, and logistic regression then classifies Design- vs. Belief-mode based on the embedding dimensions as a predictor. Thirdly, DistilBERT [25,26], fine-tuned for Design vs. Belief classification, was applied as a transformer model to contextualize word meaning and outperform other classification models. DistilBERT retains 95% of BERT’s performance while using fewer parameters, making it a viable contextual epistemic framing model. Topic-based cross-validation analysis was performed and summarized (Table 3).

ERP analysis: R Version 4.3.2 was used for statistical analysis [27]. Paired-sample *t*-tests were performed to compare N400 amplitudes elicited by Design-mode vs. Belief-mode scaffold sentences at the selected electrode sites within the 380–430 ms window. We operationalized the size of N400 as the mean amplitude between 380 and 430 ms averaged across the midline scalp site electrodes (Cp1, Cpz, Cp2, Fc1, C1, Cp1, Fc1, Fcz, Fc2, C1, Cz, C2) based on the previous literature [28,29,30].

Response analysis: Descriptive analysis and *t*-test of participants’ response data from Chronos were analyzed (Table 4).

Pre- and post-survey analysis: Survey data were analyzed using IBM SPSS Statistics Version 31.0. Paired-sample *t*-tests were performed to examine pre-post changes within each condition. 2 × 2 mixed ANOVAs were conducted to examine whether the two conditions, Design-mode vs. Belief-mode, differed significantly in their pre-post changes. False-discovery rate (FDR) correction was applied to account for multiple comparisons.

## 5. Results

### 5.1. RQ1: How Do Conversational Patterns Differ Between Two Experimental Conditions Using Design-Mode and Belief-Mode Scaffolds?

Table 1 presents the descriptive results of the KF collaborative notes for three topics for Design-mode scaffolds (My theory) and Belief-mode scaffolds (I agree/I disagree). Across all conditions, Design-mode revealed a higher number of words per note as well as a higher number of build-ons.

Table 2 shows that the SVM with the TF-IDF approach achieved a high accuracy (0.684), and DistilBert generated the highest accuracy (0.736). The classification measures accuracy and F1 scores, which indicate how well the model performs at recognizing these two scaffolds. Table 2 shows that classification using TF-IDF with SVM yields a system that performs well in recognizing Design-mode and Belief-mode discussion data. Sentence embeddings with logistic regression achieved slightly lower accuracy (0.65) but still showed appropriate predictive power in differentiating these two settings. At the same time, classification using DistilBert showed the highest prediction accuracy (0.736), demonstrating that it benefits from processing contextual data. (Table 2: Acc stands for accuracy, which refers to the total number of accurately classified notes divided by the total number of notes. F1-score refers to how accurate a model is by using the precision score and the recall score, where 2 times precision times recall is divided by precision plus recall.).

To further test the accuracy and overfitting of the models, we performed a topic-based cross-validation test, in which each folder holds out an entire topic, training on two of the topics and testing on the remaining unseen topic. The results from the topic-based cross-validation indicate that the three models are not overfitting to topic-specific vocabulary (Table 3). The TF-IDF + Linear SVM model has a mean accuracy of 0.654 (SD = 0.013) and mean F1 = 0.623 (SD = 0.010), which suggests that scaffolded-mode differences have stable lexical patterns that generalize across topics. The result of Sentence Embeddings + Logistic Regression achieved a modest result, with accuracy mean = 0.541 (SD = 0.027), and F1 mean = 0.565 (SD = 0.04). The result suggests that the semantic similarity is insufficient to capture the epistemic scaffold differences when generalizing across topics, and the slightly higher SD related to other models also suggests less stable generalization. Lastly, DistillBERT returned the highest scores with accuracy mean = 0.736 (SD = 0.01) and F1 mean = 0.705 (SD = 0.035); the high consistency scores across each fold suggest the robustness of this DistillBERT model.

### 5.2. RQ2: How DOES the N400 Differ Between Design-Mode and Belief-Mode Sentence Sets During a Stimulus-Based Decision-Making Task?

**Figure 4:**
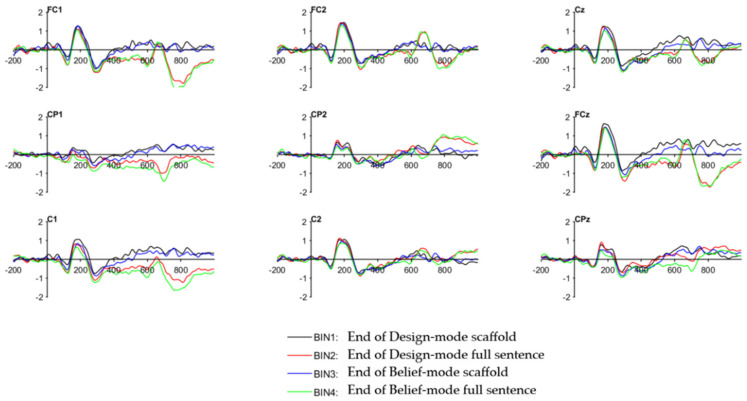
Grand average ERP waveforms for different modes: Bin1: end of Design-mode scaffolds; Bin 2: end of Design-mode full sentence; Bin 3: end of Belief-mode scaffold; Bin 4: end of Belief-mode full sentence. Measurement units: Y-axis of waveforms: μV (microvolts); X-axis: ms (milliseconds).

**Figure 5:**
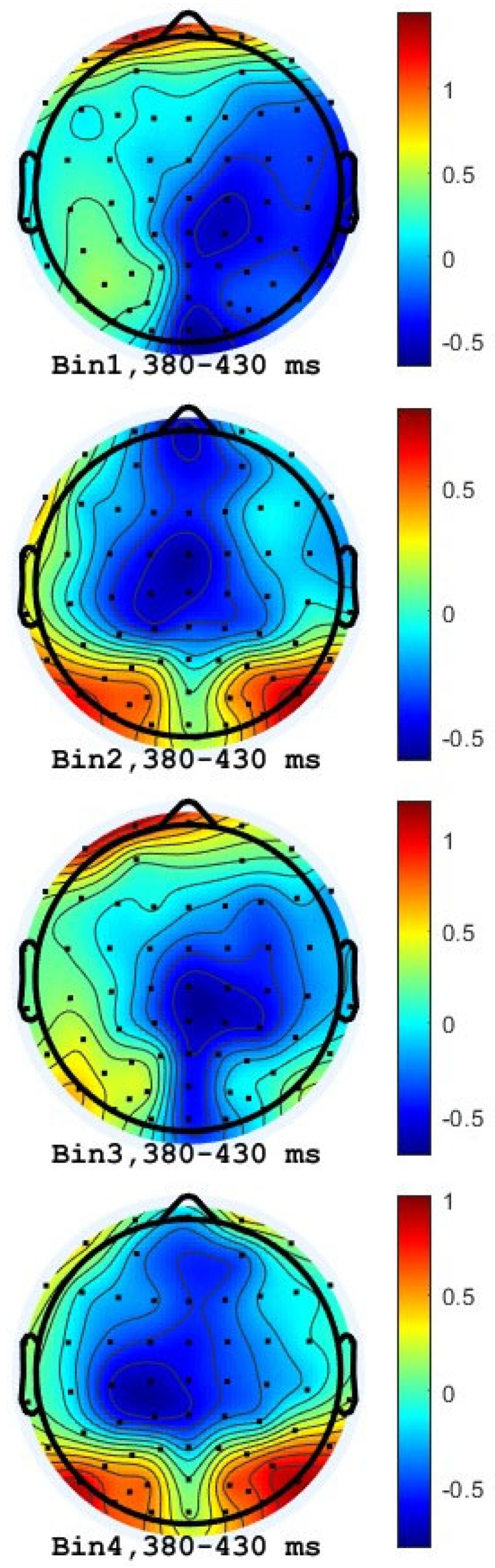
Grand average ERP. Amplitude is expressed in microvolts (μV), with negative values plotted upward. Time is expressed in milliseconds (ms). Bin1: End of Design-mode scaffolds; Bin 2: end of Design-mode full sentence; Bin 3: end of Belief-mode scaffold; Bin 4: end of Belief-mode full sentence.

Grand average ERP waveforms for the two types of scaffolds are shown in Figure 4. The data were averaged across all participants. The preliminary evidence from inspection of the ERP waveforms reveals that at the time window from 380 to 430 ms, there are statistical differences between Design-mode and Belief-mode scaffolds. Visual inspection of the scalp topographies indicated a clear N400-like negativity over fronto-central and centro-parietal regions (Figure 5). Belief-mode scaffolds and full sentence expressions showed larger negativity as compared with Design-mode scaffolds and expressions. Both Belief-mode scaffolds (Bin3 and Bin4) showed larger N400s than Design-mode scaffolds (Bin 1 and Bin 2). The region-of-interest (ROI) analysis indicated that Design-mode scaffolds (Bin1) revealed statistical significance from Belief-mode scaffolds (Bin3) in the centro-parietal ROI (Cp1-Cpz-Cp2; *p* = 0.050, d = 0.22), and in the left fronto-central-parietal ROI (Fc1-C1-Cp1; *p* = 0.034, d = 0.29). The Belief-mode sentence set showed more negative mean amplitudes in these two regions than the Design-mode sentence in the window of 380–430. Moreover, the sentence endings elicited statistically significant differences between Design-mode (Bin2) and Belief-mode (Bin4) across various symmetric and midline ROIs (including Fc1-C1-Cp1, *p* = 0.034, d = 0.29; Fc1-Fcz-Fc2, *p* = 0.036, d = 0.11; and C1-Cz-C2, *p* = 0.023, d = 0.22). In all cases, the N400 showed more negative responses for the Belief-mode sentences than for the Design-mode.

To further understand participants’ behavioral patterns from the ERP responses (Table 4), descriptive data and *t*-tests across all 52 ERP-matched participants were analyzed. On average, participants agreed with 92.7% (SD = 9.0) of Design-mode sentences, with minor disagreement and neutral responses (4.8% and 2.5%, respectively). On the other hand, Belief-mode sentences received agreement on only 59.5% (SD = 20.3) of trials, the disagreement rate increased to 36.0%, and neutral responses remained at a low percentage of 4.5% (SD = 10.5). Researchers further performed a paired *t*-test, which showed that the agreement rate was statistically significantly higher for Design-mode sentences than for Belief-mode sentences, t (51) = 11.303, *p* < 0.001, d = 1.567, illustrating that participants responded to the two sentences differently during the task. Researchers further explored how previous scaffold use during the discussion tasks influenced ERP evaluation responses (Agree/Disagree/Neutral). The results of an independent *t*-test show that participants who had used Design-mode scaffolds during the discussion task agreed with Belief-mode sentences on 53.6% (SD = 19.5), in comparison to 64.1% (SD = 19.9) among those who had previously used Belief-mode scaffolds during the discussion; t (50) = −1.917, *p* = 0.030 (one-tailed), d = 0.535. The results suggest that scaffold use during the previous discussion in Design-mode scaffolding may have generated more skepticism towards Belief-mode-framed sentences, which aligns with the interpretation that scaffold use may shape epistemic stance beyond the immediate discussion context.

### 5.3. RQ3: How Do Participants’ Attitudes Differ Between Design-Mode and Belief-Mode Conditions in Their Pre- and Post-Survey?

An independent samples *t*-test revealed no significant baseline (pre) differences between Design-mode and Belief-mode participants’ responses across survey items. Paired-sample *t*-test results for items that showed significant pre-post changes within each condition are presented in Table 5. Overall, an increased support for peer learning was observed for both conditions post-experiment. For instance, responses to the item “I enjoy working with my peers to create a shared understanding of a topic rather than just focusing on my individual learning” increased significantly post-experiment, especially in the Design-mode condition; t (31) = −5.46, *p* < 0.001, d = 0.97. More items also showed pre-post changes in the Design-mode condition than in the Belief-mode condition. To address RQ3, 2 × 2 mixed ANOVAs were conducted to examine whether the two conditions differed significantly in their pre-post responses. Results revealed no significant changes for any survey item between the two conditions after FDR correction.

## 6. Discussion

For effective collaborative conversations, scaffolds determine how the conversation starts, which in turn influences the direction and the quality of the discussion. This paper, as a proof-of-concept, examined the use of scaffolds as a language starter and its influence on participants’ discourse patterns, neural activity, and mindset changes. Results showed that machine learning models were able to distinguish participants’ discourse patterns across the two scaffold conditions at the lexical, semantical, and contextual dimensions. The ERP stimuli task further supported such differences, suggesting bigger semantic conflict produced by the Belief-mode scaffold-guided sentences. Furthermore, the pre- and post-surveys assessing participants’ knowledge-building mindset revealed more items reaching more statistically significant pre-post changes in the Design-mode condition.

Firstly, empirical research within the literacy and knowledge-building field has built a foundation between dialogically organized instruction and students’ participation patterns and understanding. Students’ use of scaffolded support, such as sentence starters in progressive discourse, is one way to shape the quality and trajectory of discourse, determine their engagement, and support the applications of conventions for advancing discussions toward a knowledge product [31]. In the field of computer-supported collaborative learning, research is often based on two traditions; social–cognitive and interpretive, where the latter often indicates a higher level of sophisticated epistemology as it seeks more explanation-seeking questions. Discussion models and patterns in KF are classifiable and meaningfully distinct. From a cognitive perspective, scaffolds in KF were designed to maintain participants’ focus on cognitive processes across three modes—knowledge sharing, knowledge construction, and knowledge creation, with knowledge creation in KF often using sentence starters such as “my theory” [32]. Along the same lines, Fu et al. [33] further expanded the three classifications into nine discourse patterns to indicate collaborative interactions and demonstrated why and how such interactions are successful or unsuccessful. The data presented suggest that the nuances of various scaffolds shape participants’ attention and are socially constructed differently during textual interpretation. The findings also align with the results of our study, which indicate that the Design-mode scaffolds show a higher build-on note rate in KF discussions.

Classification of Design-mode and Belief-mode discourses across lexical, semantic, and contextual models align with prior stance-detection research, which shows that stance is fundamentally encoded in epistemic and linguistic features. Stance determines the writer’s message and the words they choose. One of the major concerns in stance detection is inferring the embedded viewpoint from the author’s text. Since stance may not align with sentiment, the aim of stance detection is to classify the writer’s position toward a target that may be implicitly expressed in text. The stronger performance of DistilBERT relative to TF-IDF and sentence embedding is consistent with previous findings that contextualized transformer models are better at capturing the semantic features underlying stance [34], whereas traditional machine learning techniques often do not consider the contextual meaning of words. TF-IDF indicates that epistemic scaffolds introduce stable lexical regularities and detections, echoing earlier stance-detection results demonstrating that N-gram and TF-IDF features can effectively and reliably detect stance when linguistic framing is systemic [35]. Thus, deep learning models were adopted (DistilBERT) more frequently to fill such gaps. Contextual word embedding captures pragmatic and semantic features beyond surface lexical patterns. Furthermore, studies have found that most stance-detection models operate at the content level, using linguistic features [36]. However, such an approach may rely solely on textual cues and be applied across technology platforms. Thus, it further supports the view of stance as a public act achieved through communicative approaches, involving objective evaluation, subject positioning, and alignment with others. The study’s findings showed how epistemic scaffolding shifted participants’ epistemic space during the discussion. The findings from the KF online collaboration activity indicate that the use of different scaffolds shifted participants’ epistemic stance, consistent with their role in facilitating epistemic growth and epistemic space.

The ERP stimuli task further highlighted differences when participants were presented with different sets of scaffold-guided sentences, extending van Berkum et al.’s [37,38] claim that scaffolds may operate at discourse-level representation. As reported in the Results (Table 4), behavioral data and responses further supported the neural differences observed. However, such differences were only observed within the 380 and 430 ms window, in the middle and left regions. This time window is consistent with N400-like activity indexing context-related semantic processing within the typical 200–600 ms range [13], suggesting that the effects emerge at the retrieval stage, but not at the integration stage [39]. As shown in Figure 5, scalp topographies indicate that the N400 effect was distributed across fronto-central and centro-parietal regions, consistent with the typical scalp distribution of the N400 component documented in the literature [13]. The behavioral response data further provided supplementary data for neural differences observed in the ERP analysis. The strong within-subjects difference in agreement rates indicates participants engaged in different responses in the two modes of sentences, making the N400 difference unlikely to reflect a uniform decision-making strategy or general response bias. The higher disagreement rate for Belief-mode sentences further suggests that these sentences illustrate higher evaluation conflict for participants, consistent with the larger N400 amplitudes observed in the same condition.

It is also important to note that the observed differences in N400 might not reflect solely semantic conflict at the discourse-level framing or conversational implicature. Pragmatic and discourse-level differences between the two sets of scaffolds cannot be fully ruled out as contributing factors, even if the current design of the propositional content was held constant across conditions. Thus, the result is considered an early sign of diverging processing. Future research should closely regulate these stimulus elements.

The pre- and post-surveys revealed changes in attitudes before and after participation in study activities within conditions, with Design-mode showing more item-level shifts. Between-condition differences were modest, suggesting that changes in epistemic aptitude may take time. One possible interpretation is that ERP indicators are sensitive to rapid contextual modulation at the millisecond level, whereas attitudinal orientation may require repeated exposure over time. Often, mindset shifts may require sustained exposure; such dissociation is not unexpected, given that previous research has found that changes across representational levels may unfold gradually [40]. This pattern is consistent with findings that neuroplastic responses function across multiple temporal scales [41]. In summary, these findings suggest that linguistic scaffolds operate across various levels of linguistic, neural, and reflective aspects, with different temporal dynamics.

This paper also has several limitations. First, the study lasted only 2.5 h on average. Within such a short time, researchers could not measure long-term epistemic restructuring. Second, due to the online discussion activity, this study only recruited a small sample size of 66 participants. Large datasets are needed to assess robust effects of linguistic scaffolds. Third, this study tested only a small number of language scaffolds, with each pair using only one set of scaffolds in the online discussion task and each pair using only five sets of scaffolds in the ERP stimuli task. Although similar trial numbers have been reported in the literature, with ERP paradigms ranging from 6 to 400 trials [42,43], we acknowledge as a limitation that repeated exposure may have brought familiarity effects that could influence the N400, despite aligning our design with prior protocols. A larger set of scaffolds and stimulus trials with advanced statistical analysis (e.g., cluster-based analysis or mixed-effect models) could be examined in future studies to strengthen the evidence for their effects and potential differences between the two modes. Additionally, N400 amplitude has been shown to vary with age and language background, as the current study sample consisted of adult, fluent English speakers, limiting generalizability to other bilingual populations. Future research can further investigate this aspect.

## 7. Conclusions

In summary, this paper aimed to investigate how different epistemic scaffolds influenced participants’ collaborative discourse under the collaborative condition and the individual condition. Additionally, we aimed to understand participants’ attitude changes before and after the use of scaffolds. With reference to conversational patterns, machine learning models (TF-IDF + SVM, embeddings, DistilBERT) distinguished Design-mode and Belief-mode discourse across lexical, semantic, and contextual dimensions. ERP analyses revealed larger N400 amplitudes for Belief-mode scaffolds and sentences than for Design-mode scaffolds (380–430 ms), potentially indicating lower semantic fit or evaluative processing. The N400 amplitude difference between two conditions over fronto-central and centro-parietal regions is visually illustrated by grand average ERP waveforms and topographic maps. Lastly, participants showed positive changes in responsibility and collaboration, reflecting shifts in knowledge-building attitudes. Future research should examine additional scaffolds and examine ERP responses in naturalistic settings, such as face-to-face discussions.

## Figures and Tables

**Table 1:** Descriptions of Knowledge Forum notes of discussion topics.

Topic	Mode	Notes (*n*)	Words/Note Mean (SD)	Build-On Notes (*n*)	Build-Ons (%)
Topic 1	Design	203	45.22 (34.01)	118	58.1%
	Belief	214	42.75 (31.69)	108	50.5%
	Total (Topic1)	417	43.95 (32.83)	226	54.2%
Topic 2	Design	205	51.34 (35.60)	119	58.0%
	Belief	236	47.39 (32.33)	132	55.9%
	Total (T2)	441	49.22 (33.91)	251	56.9%
Topic 3	Design	239	41.93 (31.81)	148	61.9%
	Belief	248	37.27 (24.69)	149	60.1%
	Total (T3)	487	39.56 (28.47)	297	61.0%
All Topics	Design	647	46.22 (34.19)	385	59.5%
	Belief	698	42.30 (29.78)	389	55.7%
	Total (All)	1345	44.23 (32.16)	774	57.5%

**Table 2:** Results of the accuracy and F1 scores of the three methods in text classification.

Model	Overall (Acc/F1)	Topic 1 (Acc/F1)	Topic 2 (Acc/F1)	Topic 3 (Acc/F1)
TF-IDF + Linear SVM (lexical)	0.684/0.672	0.583/0.598	0.652/0.644	0.653/0.653
Sentence Embeddings + Logistic Regression	0.651/0.638	0.619/0.644	0.584/0.543	0.602/0.571
DistilBERT (contextual)	0.736/0.703	0.619/0.467	0.618/0.553	0.622/0.584

**Table 3:** Results of the topic-based cross-validation analysis, which indicates the models are not overfitting to topic-specific vocabulary.

Model	Fold1_Acc	Fold2_Acc	Fold3_Acc	Mean_Acc	SD_Acc	Mean_F1	SD_F1
TF-IDF + SVM	0.655	0.637	0.669	0.654	0.013	0.623	0.010
Embeddings + LR	0.552	0.503	0.567	0.541	0.027	0.565	0.040
DistilBERT	0.722	0.744	0.743	0.736	0.010	0.705	0.035

**Table 4:** Response rate from Chronos (Agree/Disagree/Neutral) for Design-Mode and Belief-mode sentences by groups (ERP-matched sample, N = 52).

Comparison	All N = 52 M % (SD)	Design-Mode Participant (*n* = 23) M % (SD)	Belief-Mode Participant (*n* = 29) M % (SD)	t (df)	*p*	d
1. Within-subjects analysis: agree rate for Design-mode vs. Belief-mode sentences (all N = 52, paired-sample *t*-test, df = 51; group columns descriptive only)						
Design-mode sentences						
Agree %	92.7 (9.0)	93.0 (9.1)	92.6 (9.1)	—	—	—
Disagree %	4.8 (7.8)	5.2 (7.9)	4.5 (7.8)	—	—	—
Neutral %	2.5 (5.7)	1.8 (5.2)	3.0 (6.1)	—	—	—
Belief-mode sentences						
Agree %	59.5 (20.3)	53.6 (19.5)	64.1 (19.9)	—	—	—
Disagree %	36.0 (20.9)	42.0 (21.9)	31.3 (19.2)	—	—	—
Neutral %	4.5 (10.5)	4.4 (12.3)	4.6 (9.2)	—	—	—
Agree rate: Design-mode vs. Belief-mode sentences	—	—	—	11.303 (51)	<0.001	1.567
2. Effect of prior scaffold use on Belief-mode sentence agree rate (independent *t*-test, df = 50)						
Belief-mode sentence agree rate	—	53.6 (19.5)	64.1 (19.9)	−1.917 (50)	0.030	0.535

**Table 5:** Pre-post changes within Design-mode and Belief-mode conditions. For each survey item, the first-row reports results for the Design-mode and the second-row reports results for the Belief-mode.

Survey Item	t	df	*p*	Cohen’s d
I don’t think it’s necessary to evaluate or reflect on the progress of a learning community as long as individual goals are met.	2.183	31	0.037	0.386
	0.758	33	0.454	0.130
I prefer to stick to familiar ideas and concepts rather than taking risks and exploring new directions in my learning.	2.396	31	0.023	0.424
	0.751	33	0.458	0.129
I believe that my peers and I share the responsibility for our learning.	2.436	31	0.021	−0.431
	2.510	33	0.017	−0.431
I enjoy working with my peers to create a shared understanding of a topic rather than just focusing on my individual learning.	5.463	31	<0.001	−0.966
	2.534	33	0.016	−0.435
I find providing and receiving feedback from my peers to be a crucial part of learning.	2.609	31	0.014	−0.461
	2.264	33	0.030	−0.388
I can identify which idea is promising for further investigation.	3.150	31	0.004	−0.557
	1.139	33	0.131	−0.195
I can come up with good questions.	2.709	31	0.011	−0.479
	1.997	33	0.054	−0.343

## Data Availability

The data presented in this study are available on request from the corresponding author due to ethical restrictions and participant privacy, as the data contain identifiable neurophysiological and behavioral information under Institutional Review Board approval (IRB-2024-132).
